# Proceedings of the Morgan County Medical Society

**Published:** 1866-07

**Authors:** R. E. McVey, G. R. Bibb

**Affiliations:** Pres’t.; Sec’y.


### PROCEEDINGS OF THE MORGAN COUNTY MEDICAL SOCIETY.

The Morgan County Medical Society met in the Court House, Jacksonville, at 2 o'clock P.M., May 17, 1866, pursuant to adjournment, the President being in the Chair. The minutes of the previous meeting were read and approved.

The President announced the report of the Committee on the Constitution to be in order, which was, accordingly, read by the Secretary.

On motion, it was resolved to adopt each article separately. The several Articles of Section I. were adopted without eliciting debate, but Article I. of Section II., which reads as follows:—

"Any honorable member of the regular medical profession, who is not accredited in the community where he resides as Homœopathic, Hydropathic, Eclectic, &c., also, who is a graduate of a respectable school, or of ten years' reputable practice, but if an under-graduate, he must secure a certificate of attainment from the examining board. All gentlemen thus qualified participating in this organization, by signing this Constitution and paying a fee of one dollar to the Treasurer, shall become members."

On motion of Dr. Henry Jones, it was resolved to amend, by striking out "&c." after the word Eclectic. Dr. Reed objected to the word "accredited," and moved to amend by striking it out, which was seconded by Dr. Wilber, with the explanation that he did so to give Dr. Reed an opportunity to present his objection, and not because he objected to that word himself, for he should vote against its being stricken out.

Dr. Reed proceeded to explain his objection. Said he might be accredited in this community with being what he was not, a Homœopathist; that whereas he might have expressed his conviction that there was power in attenuated medicines in certain cases, that he did not receive or practice Homœopathy as a system. It was said, in reply to the Doctor, that if he desired the coöperation and assistance of his professional brethren to disabuse the public mind in reference to his being a Homœopathist, that they would cheerfully lend their aid, that he might not thus be accredited in this community.

As the Doctor signed the Constitution and became a member, it is understood by the Society that he does not accept Homœopathy as a system of practice. The motion to amend this clause being lost, it was accepted with the remaining Articles, without further debate.

The ceremony of signing the Constitution being over, the Society proceeded to the election of permanent officers.

On motion of Dr. Askew, it was resolved to dispense with the established form, by ballot, on the present occasion, for want of time.

Dr. Askew nominated Dr. McVey, of Waverly, for President the ensuing year, which being put to the Society by the Secretary was carried by a unanimous vote.

On motion of Dr. Stephenson, Dr. J. P. Johnson, of Lynnville, was elected Vice-President.

On motion of Dr. Edgar, Dr. Bibb was chosen Secretary by a unanimous vote.

Dr. J. Craig, of Arcadia, having been nominated for Treasuser, was elected by a unanimous vote.

The following gentlemen were elected as Examining Committee:—Drs. W. S. Edgar, D. Prince, Henry Jones, C. Fisher, and J. Askew.

It being ascertained that the Society was entitled to four delegates to the State Medical Association, the following gentlemen were elected by ballot to represent the Society the present year:—Drs. Henry Jones, Askew, Miller, of Waverly, and Bibb, of Jacksonville.

The President announced the next order of business to be the reading of an essay by Dr. Henry Jones on Asiatic Cholera. The Doctor having seated himself at the desk, commenced to read his manuscript at the forty-ninth page (fearing that there would not be time to read all.) He was listened to with marked attention and interest to the conclusion, nearly two hours.

On motion of Dr. Prince, it was resolved that Dr. Jones be solicited to furnish a copy of his dissertation to some medical journal for publication. The thanks of the Society were tendered to Dr. Jones, for his very able and instructive essay.

On motion of Dr. Edgar, it was resolved to make Asiatic Cholera the subject of general debate at the next meeting of the Society. Dr. Prince suggested that Dr. Edgar open that discussion.

The following gentlemen were elected honorary members of the Society:—Drs. J. T. Cassel, Samuel Adams, and Morrison, of Jacksonville; W. B. Perry, of Berlin; James Layton, of Manchester; and Charles Chandler, of Chandlersville.

On motion, it was resolved that the next meeting of the Society should take place at 10 o'clock A.M., in this place, on the second Thursday in June, to which time the Society adjourned.

R. E. McVEY, M.D., *Pres't.*

G. R. BIBB, M.D., *Sec'y.*

